# Supplementary material for: Computational prediction of intracellular targets of wild-type or mutant vesicular stomatitis matrix protein
Source: PLoS One. 2022 Feb 2;17(2):e0263065. doi: 10.1371/journal.pone.0263065 (PMC8809538; doi:10.1371/journal.pone.0263065)
Supplement: Attachment — Submitted filename: reviewer_response.docx [file pone.0263065.s001.docx]

*We thank the reviewer for these comments. We have performed new experiments and revised the manuscript to address them as follows:*

The article by Morris et.al implements Boolean modelling to predict the targets of vesicular stomatitis matrix protein to understand the role in suppression of type-1 interferon pathway.

While the concept of implementing the logical model is reasonable, the paper lacks description. Most concepts are not described sufficiently well in the manuscript. It is important certain concepts/ model techniques are explicitly described rather than just citing papers that describe those techniques so that the article is easy to follow for even non-computational readers.

The authors perform differential expression analysis (section 2.2 ), the results for the same are not been presented.

*The extraneous portions of the differential expression analysis have been removed from the methods.*

Section 2.3 Pathway studio is used to perform text mining, however the search key word and criteria used in describing the interactions is not clearly described. It will be useful to have this information for reproducibility using open-source software as well.

*Details for the search query have been added to the methods (Section 2.3):*

*Each gene which showed significant dynamic variation over time or across viral infection by ANOVA was included in the search query as a network entity. Interactions between entities were limited to those with a documented mechanism of direct protein-protein interaction or modification (represented in the knowledge graph as “direct regulation”, “protein modification”, or “promoter binding” relations).*

Section 3.1 The discretization method doesn’t explain how the cutoff was set. Mathematical expression or textual interaction map is missing. Only a figure is provided (Figure-2)

*We have extended the description of the discretization procedure (Section 3.1):*

*The mRNA expression of each network entity was expressed as the log2-transformed fold change relative to mock-infected cells. These fold changes were discretized by projection onto two gamma-distributions using expectation-maximization to compare the measured values with the different expected distributions under assumptions of relative activation or inhibition, resulting in values of 1 or 0 for each data point [44]. The median discrete value for each combination of virus strains and timepoints was then taken to represent the activation of each gene under each experimental condition.*

Section 3.3 Biological relevance/importance of Edge fixation is not detailed.

*We have performed additional experiments to validate the interactions predicted by the process of edge fixation. The results of these experiments corroborate the novel predictions of differential regulation. We have added the following to the results (3.3):*

*To assess the accuracy of these novel predicted interactions, we additionally measured the transcription of TNFAIP3 in L929 cells infected by 22-20 or 22-25 VSV via RT-PCR. While both viruses significantly upregulated TNFAIP3 transcription as predicted by the model (Fig. 6), there was slightly significant statistical evidence that the magnitude of this upregulation was lower in cells infected by 22-25 (p<0.1)*

*as well as the Discussion (4):*

*The strain-specific regulatory interactions predicted by the computational modeling procedure are corroborated by the confirmatory RT-PCR we performed to measure TNFAIP3 transcription in infected cells. While both 22-20 and 22-25 strains upregulated TNFAIP3 relative to uninfected cells, resulting in their converging to similar values on a binary scale, we observed greater upregulation by 22-20 VSV, consistent with the differential regulation of TNFAIP3 by these strains predicted by our model.*

*and an additional figure (Figure 6), with the following caption:*

*Figure 6. Differential regulation of TNFAIP3 by 22-20 and 22-25 VSV. The log2 fold change relative to uninfected (Mock) cells is displayed. TNFAIP3 mRNA is significantly upregulated by both 22-20 and 22-25 relative to Mock (p<0.05), and the magnitude of upregulation is greater in 22-20 (p<0.1).*

Better description of PCA analysis is required.

*We have added to the description of the PCA results (Section 3.1):*

*PCA performed on the significantly variable genes was able to explain some 88% of the total variance with the top 2 components. With respect to these 2 components, all 3 viruses strongly diverge from the mock samples by 3 HPI, though the 22-20 virus did not appear to induce substantial transcriptional activity by 1 HPI (Fig. 1). The results of the PCA show substantial differences in the transcriptional response of L929 cells to each VSV strain, demonstrating the necessity for interrogation of the regulatory dynamics of these responses.*
